# Cultivating long-term well-being through transformative undergraduate education

**DOI:** 10.1093/pnasnexus/pgae372

**Published:** 2024-09-24

**Authors:** Holly C White, Debra M Allen, Keith Buffinton, Dana Humphrey, Marjorie Malpiede, Richard K Miller, John C Volin

**Affiliations:** School of Biology and Ecology, University of Maine, Orono, ME 04469, USA; Office of Institutional Research and Assessment, University of Maine, Orono, ME 04469, USA; College of Engineering, Bucknell University, Lewisburg, PA 17837, USA; LearningWell Magazine, Boston, MA, USA; LearningWell Magazine, Boston, MA, USA; Franklin W. Olin College of Engineering, Needham, MA 02492, USA; Office of the Provost, University of Maine, Orono, ME 04469, USA; School of Forest Resources, University of Maine, Orono 04469, ME, USA

## Abstract

Despite the growing body of research suggesting certain pedagogical approaches that can support student well-being, higher education has not fully embraced these approaches and typically still does not view well-being as a high priority in comparison with other metrics such as retention or GPA. Here, we contend that universities must play an active role in supporting lifelong well-being in their student populations by expanding their definitions of student success and providing opportunities and programs that support elements related to well-being. We propose a student well-being nexus, which comprises a sense of belonging, agency, purpose, identity, civic engagement, and financial well-being. This article provides a perspective on the importance of each element to well-being and which pedagogical practices have been shown to support various dimensions of well-being in undergraduate education, such as service learning, undergraduate research, and mentoring. In addition, it showcases 6 exceptional initiatives from various universities that aim to support one or more of the student well-being nexus dimensions, which can serve as models for other universities. Finally, several guiding principles are outlined for higher education institutions to support the implementation of student well-being initiatives and transformational learning opportunities. These include assessment of initiatives, embedding initiatives into the curriculum, and avoidance of creating additional financial burdens for students. These efforts can promote well-being on college campuses and beyond graduation.

## Introduction

There is a growing body of research that shows that certain types of pedagogical methods in higher education positively influence student well-being outcomes over time. These transformational experiences include activities, approaches, and relationship dynamics that engender belonging, identity, purpose, and agency in students, the obtainment of which has been proven to increase overall well-being, among other factors. But despite the correlating evidence and existence of best practices, higher education has been slow to embrace these methods and continues to rely on traditional measures of success, mainly with metrics such as retention and graduation rates (1), GPA, and grades (2). Further, undergraduate students face society and family pressures to achieve extrinsic goals such as material success, wealth, and status rather than intrinsic goals such as happiness, well-being, and meaning [e.g. (3, 4)].

Higher education can play a critical role in supporting students to achieve these intrinsic goals associated with lifelong well-being. A long-term Gallup study found that 6 key college experiences are linked to preparedness and well-being later in life (5): (1) having at least 1 professor who makes you excited about learning, (2) having professors who care about you as a person, (3) having a mentor who encourages you to pursue your goals, (4) working on a project that took a semester or more to complete, (5) having an applied internship or job, and (6) being extremely active in extracurricular activities and organizations during college. However, few alumni (3%) actually report undergoing all 6 key experiences. Having at least 1 professor who makes you excited about learning (63%) is the most common of the 6 experiences and notably more common than the other 5 experiences, which ranged from ∼20% to 32% of alumni. The number of experiences reported by alumni was positively correlated with feeling prepared for life and having a greater sense of belonging. Expanding beyond alumni, Gallup and the Coalition for Transformational Education have now collaborated to measure these experiences among current undergraduate students. The results of the Gallup/CTE survey, which were piloted at 12 colleges and universities over a 2-year period, show that the 6 experiences are also positively correlated with well-being for current students (CTE/Gallup survey, unpublished data). This work is critical to both understanding and predicting college students’ lifelong well-being and career satisfaction.

Here, we posit that colleges and universities must take an active role in ensuring students have the opportunities to engage in pedagogical activities that have the potential to foster their long-term well-being and happiness. To do so, higher education needs to design opportunities based on prior research of experiences and programs that foster well-being, including belonging, agency, sense of purpose, identity, and civic engagement. To enable and sustain these changes, higher education must expand its definition of success to include well-being outcomes across multiple dimensions, representing the student more comprehensively.

## What is lifelong well-being?

Well-being has been defined and measured in various ways. The Gallup definition of well-being consists of 5 dimensions: (1) physical (health and energy), (2) social (strong relationships), (3) financial (managing finances to reduce stress and increase security), (4) career (liking what you do each day), and (5) community (engaging in the area where you live) (6). It has been used for more than 50 years to monitor well-being and happiness in many nations across the globe (6). The University of Pennsylvania's PERMA metric considers positive emotion, engagement, relationships, meaning, and accomplishment in their measurement of well-being (7). Harvard's Human Flourishing Program metric measures well-being by capturing happiness, mental and physical health, meaning and purpose, character and virtue, close relationships, and financial stability (8). The Gallup metric, which overlaps the others in substantial ways, has the benefit of 50+ years of historical data across the globe for populations well beyond the college years.

**Fig. 1.**
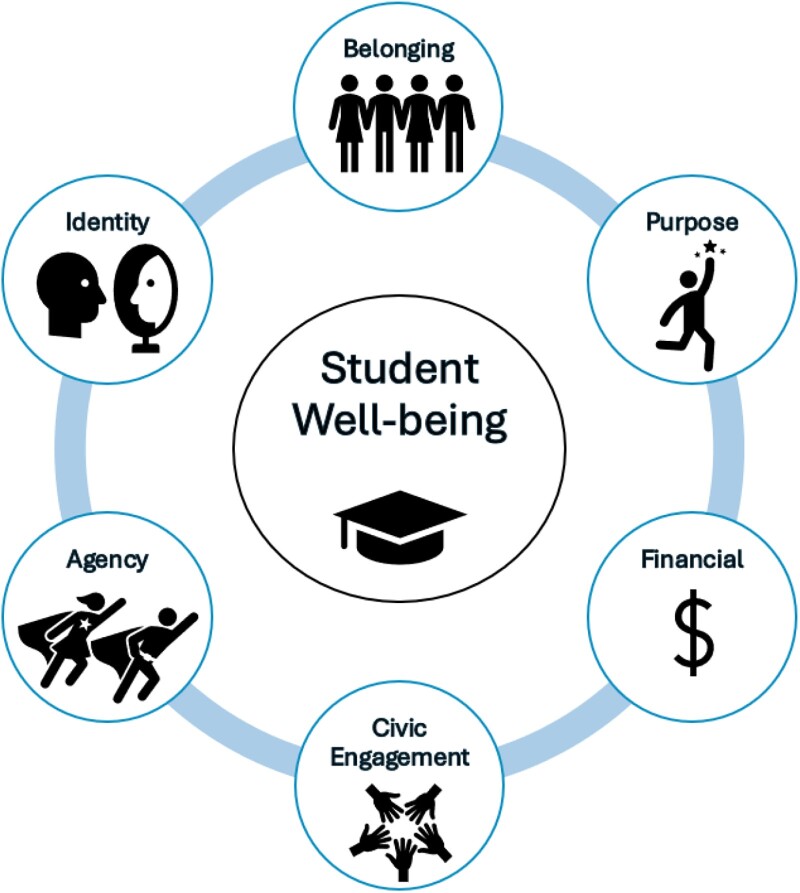
The student well-being nexus.

Here, we conceptualize student well-being as the development of and interconnections between sense of belonging, agency, purpose, identity, civic engagement, and financial well-being (see Figure 1), all of which are powerful drivers of lifelong well-being, and have been long-studied as promising in the field of education research [e.g., (9–14)]. Measuring the combined impact of these constructs to represent well-being is complex and has been approached in different ways. Belonging, or the feeling of being a part of a community (15), has been extensively studied in higher education settings to investigate retention and student outcomes (13, 16–19). A recent study by Fischman and Gardner (11) at Harvard Project Zero, based on 2,000 interviews at 10 universities across 7 years, concluded that belonging is the most common concern of college students in America today (11). Importantly, students’ perceptions of what it means to “belong” differ based on demographic variables, including socioeconomic status and gender (20).

Agency refers to the idea that one can change and contribute to their life circumstances (21), and it is particularly important for young adults as they begin to navigate their place in the world (22). Students should feel that they can influence their educational, career, life, and future trajectories (23). To have agency is to engage in intentionality, forethought, and reflexivity, or to be a proactive contributor, rather than an onlooker in one's experiences (21). University experiences that foster a sense of agency can also encourage student development of identity and overall well-being (9, 10).

To have purpose is to create and accomplish goals that are both personally meaningful and important beyond one's self (24). Purpose, or meaning in life, can develop through experiences doing something of personal or public significance, through social relationships, and through experiences coping and overcoming hardship (25). Purpose is associated with adolescent and adult well-being (26–29), greater grit among college students (14), higher household income (30), and greater ability to handle life stressors (31).

Identity refers to one's answer to the question, “Who are you?” often in relation to their characteristics, affiliations, experiences, worldviews, and social roles (32). This is complex given the potentially numerous identities (e.g. academic identity, ethnic identity), both personal and social, that one may hold (32). College is a time when students are exploring their identities (12). Consequently, a growing number of higher education initiatives focus on facilitating that exploration [e.g., (33, 34)].

Civic engagement, another critical component of the student well-being nexus, refers to “the ways in which citizens participate in the life of a community in order to improve conditions for others or to help shape the community's future” (35). Higher education plays a critical role in ensuring that students are prepared to lead lives as active, global citizens (36). Civic engagement exists on a continuum, and ranges from community- or politically oriented activities (37), which may occur at the individual level, or at public, collective level (e.g. helping a neighbor vs regular and intensive community service) (35).

Financial security is also critical to student well-being. Financial stressors for college students range from living paycheck to paycheck, lack of a safety net to cover emergency expenses, and student loan debt (38). High financial stress among college students is related to a lower perceived well-being (38) and a higher likelihood of discontinuing studies (39). Underrepresented students may face additional barriers to accessing resources to achieve financial well-being (40). Together, sense of belonging, agency, purpose, identity, civic engagement, and financial stability all play a role in lifelong well-being, and as such, it is important that higher education institutions put in place best practices that actively promote and foster them.

## Supporting lifelong well-being in undergraduate education

The Gallup alumni survey (5) and other prior research reviews (41) illustrate that (1) supportive mentors and (2) experiential or authentic learning opportunities are positively related to long-term well-being. Supportive mentors are positively related to student agency (42), sense of belonging [e.g., (43, 44)], purpose (28), identity development (45), and civic engagement later in life (46). Mentoring relationships may be particularly beneficial to marginalized students (47), and when mentors and mentees have demographic similarities (45). Whether through mentoring or not, fostering a growth mindset in both students and faculty also has the potential to improve learning outcomes for all students (48), and particularly for marginalized students (49). For example, normalizing adversity and discouraging beliefs that attribute academic struggles to fixed characteristics of themselves or social group (e.g. gender, race) can support well-being for marginalized students (16–19). Experiential and authentic learning have also been shown to have an array of positive short- and long-term outcomes (50). Further, evidence suggests that authentic learning experiences support development of STEM identity (51) and fostering identity development is an outcome of service learning (52, 53) and personal narratives and storytelling (54, 55). These experiences, if designed in a manner to do so, also play an important role in promoting student agency and autonomy (52, 56) and sense of belonging [e.g., (57)]. Other types of experiential learning are also beneficial. For example, problem-based learning (58) has been shown to foster identity development (59, 60), and design-based learning can foster agency among students [e.g. (10)]. Service learning may lead students to consider civic responsibilities in their future careers (61), and similarly, community-based projects are positively related to students’ civic engagement later in life (46). Providing opportunities to develop personal narratives and storytelling has also shown to be related to identity development (54, 55). Furthermore, racial/cultural awareness workshops (62) and dialogue with others across differences (63) have been shown to positively affect civic mindedness and engagement after college. It is also important that financial need be considered when designing interventions to support student well-being. Many research and internship experiences are unpaid volunteer positions that are inaccessible to students in financial need, who often must work outside of class to support themselves (64). Though it may not be possible to fully address financial concerns, it is critical to provide opportunities that support well-being without creating an additional financial burden for students.

## Exemplar efforts to support well-being in undergraduate education

Many institutions are making explicit commitments to embedding well-being in the undergraduate curriculum through supporting belonging, agency, purpose, identity, and/or financial well-being. We highlight several exemplary efforts here. These efforts, which represent an array of institutional categories, range from small to large in scale and from first-year to senior-level interventions. These initiatives vary greatly in approach; however, they are all seeking to make measurable impacts on their students’ current and future well-being, typically with limited resources and little national visibility.

### Purposeful work at Bates College

Purposeful Work is a program designed to help students align who they are with what they do in life by providing opportunities for them to explore their interests, identities, and strengths. The curriculum includes a first-year seminar focused on purpose and work, practitioner-taught courses for students to learn practical skills in a field, and a course for juniors and seniors who are still unsure of their plans after graduation. The program also involves internships, job shadowing, industry-specific resources, and support for those interested in graduate school. Students intentionally reflect on their experiences as they move through the program. Further, Bates College offers financial assistance to students to participate in unpaid internships, to travel for immersive experiences, and for professional attire, graduate school application fees, and interview transportation costs. College graduates with a high sense of purpose were 10 times more likely to have a high well-being (65). Further, 99% of the spring 2022 graduates reported being settled by December 2022.

### Digital storytelling at the University of Michigan–Dearborn

In 2021, the University of Michigan–Dearborn launched a digital storytelling project, More Than A Single Story: UM-Dearborn Speaks (66). This project has allowed students to gain experience working with media and technology while also exploring and reflecting upon their identities, values, and purposes. This project is particularly important because of the high Arab-American enrollment and the surrounding Muslim-American community. Students reported that the project helped them to build community, reflect, and gain storytelling skills. In this model, the students have the opportunity to mentor the following cohort that joins the project, allowing them to further build community while gaining mentorship skills.

### Research Learning Experiences at the University of Maine

Research Learning Experiences (RLEs) are course-based research experiences for first-year students in which students engage in authentic research and exploration. The program was piloted in fall 2021 and has since served 1,000+ students spanning disciplines from geology to art. The core characteristics of the courses in the program are small cohort, summer bridge immersion experience, and discovery, knowledge creation, experiential learning, and/or active learning. The program encourages peer relationships and sense of belonging, and fosters agency, purpose, and identity through enriching research experiences. Preliminary results indicate that RLE students report significantly higher on survey items including research identity, reflection, and sense of belonging to the university. The cost of the RLE plus the summer bridge experience was the same as any other credit-bearing course, and scholarships were made available for students who stated that the cost would impede their participation.

### Quest at the University of Florida

Quest aims to foster intellectual curiosity among students by encouraging them to think critically and become informed and thoughtful decision makers and citizens. While many schools share this aim, the Quest program makes it foundational by centering it within the University of Florida's general education program. Over 4 years, Quest utilizes active pedagogical approaches, rather than coursework centered around lecturing, rote memorization, and standardized testing. Specifically, students partake in 4 “quests” over their undergraduate career: (1) engaging with essential questions from the humanities (e.g. identities, justice and power), (2) engaging with essential questions from natural and social sciences (e.g. biological, behavioral sciences), (3) engaging in the world (e.g. community service, research, internship), and (4) a senior capstone project.

### Design your life at Stanford

One approach to developing students’ agency is Stanford's “Designing Your Life” course, which teaches students how to intentionally make decisions about their lives and vocations [e.g. (10, 67)]. This course, offered through the School of Engineering, applies a design thinking approach to students’ decisions regarding their life and career. Though this class is offered in the engineering department, it is for any junior or senior who wishes to think creatively and critically about their future. The class uses experiential learning and mentoring to help students design a postgraduation plan.

### Civic and ethical engagement at Wake Forest

Wake Forest supports students in becoming ethical and effective leaders through incorporating community service, civic engagement, and character development into their curriculum. Through tailored coursework, discussion groups, workshops, and community service, Wake Forest aims to prepare students to serve humanity. Research on the program is ongoing and is exploring the impacts of the program's varying pedagogical approaches on student outcomes related to civic mindedness and character.

## Guiding principles for implementing well-being initiatives

Though each initiative varies in scope, scale, and approach, there are commonalities between them that are key to fostering well-being:

1. Well-being should be embedded into the undergraduate curriculum in order to reach a wide range of students, rather than only through extracurricular or cocurricular activities, which may be costly.
2. Initiatives do not necessarily have to aim to foster all components of lifelong well-being immediately, as most successful initiatives begin by focusing on 1 or 2 areas. This targeted approach allows for a more manageable and detailed development of the chosen areas.
3. Approaches should be tailored to an institution's specific student body and university culture. Digital Storytelling at the University of Michigan–Dearborn is an example of an initiative excelling in this tailored approach.
4. Initiatives should be measured through an iterative assessment strategy on the front end that can be used to inform program changes and the scaling up of successful initiatives.
5. Given the influence of faculty, and the importance of embedding strategies into the learning environment, faculty leadership and their buy-in are important considerations.
6. Opportunities should not create an additional financial burden for students to ensure accessibility.

## Future directions

As shown here, there are an array of pedagogical strategies utilized across institutions to develop and support belonging, agency, purpose, identity, civic engagement, financial stability, and overall lifelong well-being. There is a need to unify these ongoing efforts, which the Coalition for Transformational Education is currently addressing by bringing together colleges and universities dedicated to cultivating well-being and facilitating the exchange of best practices that scale to all students and ongoing assessment. However, there is still more research needed to better understand how universities can create coordinated opportunities for undergraduate students to develop lifelong well-being. Specifically, a review of the existing programs, including the ones discussed here, would benefit our understanding of how other institutions may create similar long-lasting efforts to support students. Questions that should be addressed include the following: How much funding is needed for varying degrees of programmatic support? How are decisions made for program design? How are programs and interventions being assessed and evaluated? When considering future efforts to design similar programs, decisions should be informed by data collected during the pilot phase and continuous assessment and program evaluation.

Future research efforts include assessing immediate effects of interventions and comparing them with the Gallup alumni cohort. Specifically, research should identify what pedagogical approaches of a program can foster which elements of well-being (e.g. does a service learning intervention affect both purpose and belonging, or just purpose, and how do various interventions differ?). Research should also assess student outcomes based on other program characteristics including duration (4-year program vs semester) and targeted student group (e.g. first-years, seniors, or all cohorts). Addressing these questions will be essential as more universities begin to create similar efforts.

Research should also explore theories of change for higher education that can guide the efforts for a coalition of like-minded institutions leading efforts that support well-being. It is critical to consider context, underlying assumptions, outcomes, interventions, and indicators of success as universities connect and collaborate to transform undergraduate education (68). For example, what “theory of change” for higher education might guide the efforts for a coalition of like-minded institutions? What is the role of university leadership, faculty, staff, alumni, governance, experimentation, pilot programs, and assessment [e.g. (5)]? The current model for institutional change involves (1) validation and endorsement by campus leadership at the highest level; (2) faculty-driven interventions that address belonging, agency, purpose, and identity, aimed at every enrolled student; (3) targeting not retention, graduation rate, starting salary, etc., but instead well-being in multiple dimensions, including social mobility, long after the college years; and (4) commitment to formal assessment of outcomes and continuous adjustment and improvement of programs. It is critical to avoid focusing solely on individual change (e.g. classroom, instructor level) and to use change theory to inform research and interventions, rather than using it superficially (69). This systematic approach can ultimately transform undergraduate education using pedagogical best practices to support student long-term well-being through a focus on sense of belonging, agency, purpose, identity, civic engagement, and financial well-being.

## Concluding remarks

It is imperative that universities prioritize student well-being, which will require adoption of transformative pedagogical practices as a step toward the ultimate goal of improving well-being and flourishing long after the college years. Despite a wealth of evidence illustrating the importance of belonging, agency, purpose, identity, and financial stability to lifelong well-being (70), these are often not the focus of universities’ student success metrics or their interventions. However, this perspective piece describes initiatives such as Purposeful Work at Bates College and RLEs at the University of Maine that can serve as models for other universities. Transformative educational experiences informed by collaborations, research, assessment, and theories of change offer great potential for integrating well-being into undergraduate curriculums.

## Data Availability

There are no data underlying this work.
